# Driving Circuitry for Focused Ultrasound Noninvasive Surgery and Drug Delivery Applications

**DOI:** 10.3390/s110100539

**Published:** 2011-01-07

**Authors:** Munir M. El-Desouki, Kullervo Hynynen

**Affiliations:** 1 Imaging Research, Sunnybrook Research Institute, Toronto, ON M4N 3M5, Canada; E-Mail: mdesouki@sri.utoronto.ca; 2 Department of Medical Biophysics, University of Toronto, Toronto, ON M4N 3M5, Canada

**Keywords:** beam-steering, focused ultrasound, high-intensity focused ultrasound, hyperthermia, integrated circuits, noninvasive surgery, phase shifter, phased arrays, phased-locked loop, power amplifier, pulser, tissue ablation

## Abstract

Recent works on focused ultrasound (FUS) have shown great promise for cancer therapy. Researchers are continuously trying to improve system performance, which is resulting in an increased complexity that is more apparent when using multi-element phased array systems. This has led to significant efforts to reduce system size and cost by relying on system integration. Although ideas from other fields such as microwave antenna phased arrays can be adopted in FUS, the application requirements differ significantly since the frequency range used in FUS is much lower. In this paper, we review recent efforts to design efficient power monitoring, phase shifting and output driving techniques used specifically for high intensity focused ultrasound (HIFU).

## 1. Introduction

High-intensity focused ultrasound (HIFU) surgery allows energy to be focused deep in the body inducing noninvasive local temperature elevation that destroys the targeted tissue, while sparing the surrounding tissue. The temperature elevation can be further enhanced using cavitation bubbles [1]. Cavitation can also be used to mechanically disintegrate the tumor tissue without macroscopic temperature elevation [2]. This noninvasive method can ablate tumors without the side-effects and complications associated with invasive surgery [3]. Since magnetic resonance imaging (MRI) can provide noninvasive temperature maps in real-time, the combination of MRI and HIFU has proven to be very effective in the treatment of tumors [3]. MRI-guided HIFU has been studied for thermal ablation of pathological tissue, local drug delivery using thermosensitive micro-carriers and controlled transgene expression using thermosensitive promoters [4]. Based on these experiments, MRI-guided HIFU has shown promise in allowing complete noninvasive tumor destruction and several thousand patients have been treated worldwide for uterine fibroids and bone tumors, with ongoing development in prostate, breast and other tumor treatments [5]. MRI-guided HIFU could also be particularly beneficial for treating brain disorders and transcranial applications, as it is noninvasive, does not cause bleeding, and does not damage surrounding tissue [6]. Transcranial therapy may be used for the temporary disruption of the blood-brain barrier (BBB), which will allow for delivering therapeutic agents and drugs to the brain [7].

Over the past few decades, researchers have come up with various ways to achieve automated power control and monitoring, electronic beam-steering and focusing, automated phase control, and efficient multi-element control and driving. However, as systems continue to grow in complexity and the demands to reduce system cost, weight, and size increase to allow for wider spread and portability, researchers are continuously trying to improve the existing power and control techniques. In this paper, a review of the existing driving and control circuitry used for HIFU and phased array systems is presented. The various techniques used for phase shifting and output driving are discussed, in addition to some suggested areas of improvement.

**Figure 1.**
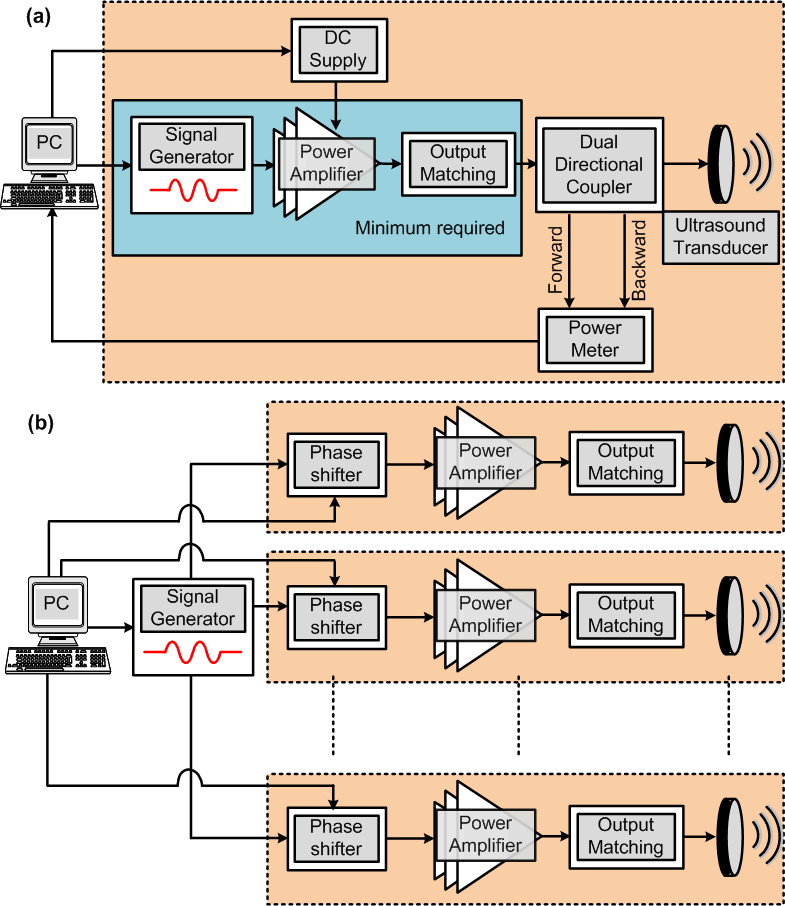
**(a)** A block-diagram of a typical single-element HIFU system. If used in an *n*-element system, the area enclosed in the dashed line should be repeated *n* times. **(b)** A more practical multi-element implementation that employs phase shifters.

Figure 1(a) shows a typical block diagram of a HIFU system (not showing the MRI control). Once the desired signal is selected using the signal generator, which is usually set in the range of 0.5 to 20 MHz, with an amplitude below 1 V, the power amplifier boosts the signal to the desired output power level, which is usually in the range of 50 to 100 W. Ensuring that the power amplifier has a high efficiency is very important since it is the most power consuming block of the system and any wasted energy will result in heat generation [8,9]. When dealing with high output power levels, it is also important that the output power be monitored and controlled. This can allow the system to immediately shut down if the output power exceeds patient safety levels. The backward power levels can also be monitored if the load is not perfectly matched. Once this system is used for more than one element, in the case of electronically focused phased-arrays for example, each transducer will require its own set of equipment, resulting in a very complicated and inefficient implementation. Figure 1(b) shows how phase shifters can be used to reduce the system complexity allowing the use of only one signal generator. This is a simplified diagram that does not show additional components such as phase monitoring and control for example. In this paper, the various blocks shown in Figure 1 will be discussed and research efforts to implement efficient HIFU systems will be reviewed. The paper is organized as follows. In Section 2, the output power monitoring and electrical to acoustic conversion efficiency are discussed, followed by the different phase shifting techniques in Section 3. The output and driver stages are discussed in Section 4, which are followed by the conclusions in Section 5.

## 2. Output Power Monitoring

Unlike ultrasound imaging, HIFU may present risks to the patient if improperly used due to the high power levels required for tissue ablation [10]. Fleury *et al.* [10] recommended using a failure mode and effect analysis (FMEA), when designing HIFU systems. FMEA requires the identification and description of failure, analyzing the risks, and defining prevention plans. Improper monitoring of the output power can result in over- or underexposure of the patient to HIFU. Underexposure, although not harmful to the patient, may result in treatment failure. It is mainly caused by inaccurate estimation of the electrical to acoustic conversion efficiency, which can be a result of poor coupling at the skin interface, or even due to transducer aging. The transducer’s efficiency should be carefully characterized by measuring the acoustic output power in reference to the input electrical power (readers should refer to [11,12] for more information on measuring the acoustic power). The efficiency should also take into account electrical losses in array cables, which could be as high as 50% [13].

Overexposure is usually a result of increased, uncontrolled electrical power being applied to the transducer. This can result in lesions larger than expected as well as an increased likelihood of cavitation. Over exposure can also result in overheating the transducers, which may lead to destruction of the transducer or a drop in its impedance [10]. A drop in impedance will result in an increase in output power. If more than one element is driven from a single amplifier a change in impedance or a failure occurring in one of the elements can result in a significant change in the output power transmitted per element. Using simple calculations for parallel impedances, the following equation can be used to calculate the increase in output power (Δ*P*):

(1) $$\Delta P=\frac{d}{n\left(1-d\right)}$$

where *d* is the drop in the impedance of the failed element, and *n* is the number of elements in the array. For example, a 50% drop in the impedance of an element in a group of 8-elements will result in an increase of 12.5% in output power. This is especially important since tissue necrosis has a logarithmic temperature dependency [14,15].

**Figure 2.**
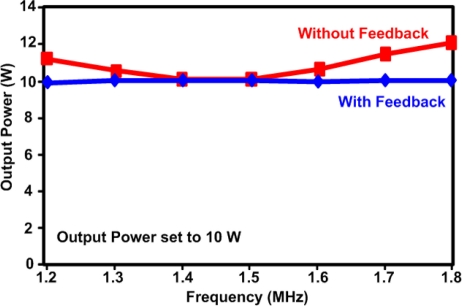
The output power applied to a 50 Ω load with and without feedback, reproduced from [15].

In some phased array designs, elements of different sizes maybe used [15]. This is another reason for monitoring the output power since it is load dependant. Power monitoring and feedback is also important to compensate for amplifier nonlinearities, which are common in the power amplifier topologies that are used for HIFU, as will be discussed further in Section 4. As shown in Figure 1(a), both the forward and reflected powers are monitored. Monitoring the reflected power can give an immediate indication of whether or not a single transducer has failed, since there will be a sudden mismatch and increase in reflections for the failed transducer [15,16]. Monitoring the reflected power also indicates how accurately the delivered output power can be measured using the forward power. Figure 2 shows an example of how regulation of the output power applied to a 50 Ω load has lowered the maximum error from 20% down to 1% [15].

**Figure 3.**
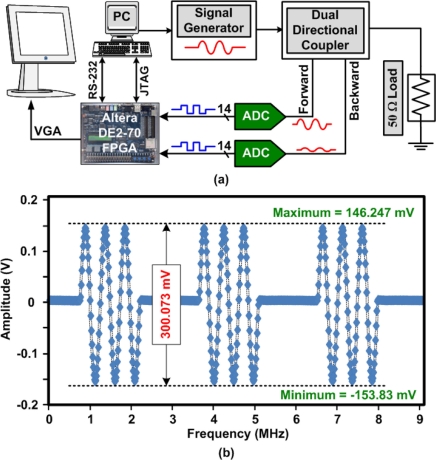
**(a)** FPGA based power meter block diagram. **(b)** An example of the sampled forward voltage waveform of a 2 MHz signal applied in a burst of 3 pulses with a burst period of 3 μs.

Although the frequencies used for HIFU are not very high (<20 MHz), the design of the power meter may suffer from bandwidth limitations that can result in inaccuracies in the power level measurements. This is mainly due to the signals being applied in short bursts. In order to accurately measure the power of a non-continuous waveform, especially when using burst periods below 10 μs, high sampling rate analog-to-digital converters (ADC) controlled by a high frequency microcontroller or field programmable gate array (FPGA) maybe required. Figure 3(a) shows a block diagram of an example of an FPGA based power meter. The system uses two 14-bit ADCs (AD9248) from Analog Devices (Wilmington, MA, USA) that are controlled with a sampling rate of 65 MHz using the FPGA (Altera Cyclone II 2C70) that is mounted on a DE2-70 evaluation board. The FPGA is also in charge of optionally displaying the waveform on an LCD monitor as well as uploading a sampled waveform to the PC through the serial RS-232 port. The system was tested by applying a 300 mV peak-to-peak, 2 MHz, sinusoidal waveform in burst mode. The output forward voltage waveform that was uploaded to the PC is shown in Figure 3(b), for an input with a short burst period of 3 μs. After the amplitude of the signal is obtained by the FPGA, it is easy to then calculate the power level in the FPGA based on the known load value.

## 3. Phase Shifting for HIFU Phased-Arrays

**Figure 4.**
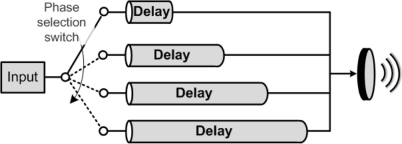
A simple phase shifter using switchable delay lines.

Phased arrays are mainly used in HIFU to allow for electrical steering of the focal point or create multiple focal points. They can also be used to enlarge the focal point [17]. The simplest way to drive a multi-element phased array will be use one of each block shown in Figure 1(a) per transducer. However, this will be very costly and inefficient especially for large arrays. Having a controllable phase shifting technique will allow for a great reduction in the number of amplifiers used. Unfortunately, the simple phase shifting techniques, such as the one shown in Figure 4, that are suitable for microwave phased arrays cannot be used for HIFU systems. In this case, the difference in delay lines is inversely proportional to the frequency of the signals and at the operating frequencies of HIFU, practical phase differences will be below 2°.

**Figure 5.**
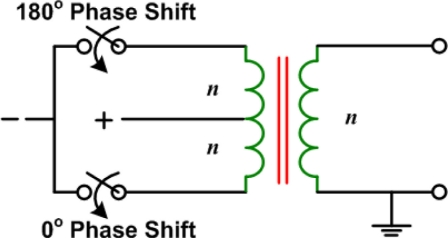
Isolation transformer used to create 180° phase shifts [17].

A two phase switchable system was proposed by Fjield *et al.* [17] that allows for reducing the number of amplifiers needed. The system uses isolation transformers, as shown in Figure 5, to produce 180° phase shifts. This can only be used for certain array geometries such as an eight-element sector-vortex array that can be driven to give a total of eight peaks or one peak and eight different field patterns using only 180° phase shifts [17].

**Figure 6.**
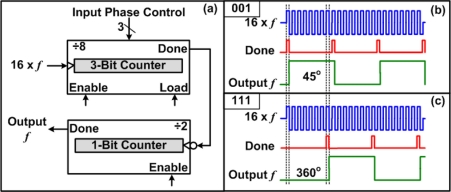
Digital phase shifter using programmable counters. **(a)** Schematic diagram, and functional operation examples of **(b)** a 45° phase shift and **(c)** and 360° phase shift.

A controllable phase shift can also be obtaining using purely digital techniques. Figure 6 shows the concept of a digital phase shifter that uses programmable counters, similar designs have been used in [15,18,19]. An example of a 3-bit digital phase shifter is shown in Figure 6(a). By using a reference clock that has a frequency that is 16 times the desired output frequency, a programmable count up counter can be used to achieve a variable phase shifter with steps of 45°. When the count up counter reaches the preloaded phase control value, the “Done” signal goes high for one clock cycle, which can be seen from Figure 6(b). The “Done” signal is then connected to a 1-bit counter (divide by 2), with an active low clock input. The 1-bit counter is used to convert the duty cycle back to 50%. Figures 6(b) and (c) show examples of 45° and 360° phase shifts that can be obtained by inputting phase control values of 001 and 111, respectively.

**Figure 7.**
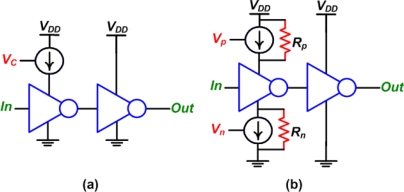
Inverter based digital delay lines. **(a)** Rise time control, reproduced from [18], and both rise and fall time control, reproduced from [22].

The main drawback of the digital phase shifter, shown previously in Figure 6, is that a multiplied reference clock is required [15,18,19]. For a phase control with a 10-bit resolution and an output frequency of 2 MHz, a 2 GHz reference clock is required. This makes achieving digital high resolution phase control too expensive and unpractical if high phase resolution is needed. In any focused ultrasound application a lower phase resolution is adequate [20] reducing the reference signal frequency to a more practical range, as will be explained later. Another technique to achieve digital phase control is to use controllable digital delay lines [15,18–21]. The simplest digital delay line uses a number of sequential delay elements. The most basic delay element uses a current starved inverter followed by a regular inverter. Figure 7(a) shows the delay element used in [18], while Figure 7(b) shows the delay element used in [22]. The advantage of the implementation shown in Figure 7(b) over the one shown in Figure 7(a) is that it offers more control since both the rise time and fall time can be changed. Also, a weak pull up resistor (*R_p_*) and a weak pull down resistor (*R_n_*) were used to ensure that the inverter is still on, even if no control voltage is applied. The resistors, as well as the current sources, were implemented using active devices with constant gate potentials. Both of these designs are suitable for CMOS integration and are compact, however, they require an analog voltage for phase control, which might give rise to error. A delay locked loop was used in [18] to stabilize the control voltage (*V_c_*).

**Figure 8.**
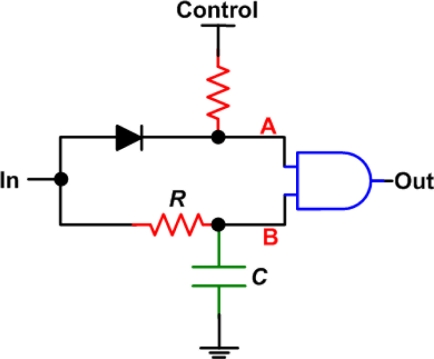
AND gate delay element, reproduced from [19].

In [19], an AND gate was used with an RC delay line (Figure 8) in order to chose between two delay values per delay element as a signal propagates through a delay chain. When the control is low, the output rises after an *RC* time constant, when both A and B are high. However, the output drops immediately following the input. When the control is high, the output still rises after an *RC* delay, however, it remains high for a period of an *RC* time constant after the input goes to zero, which gives the delay. The advantage of this circuit is that it uses a digital pulse, however, the circuit is larger and requires more components. Also, it is most likely that the capacitor used will be large and inefficient for full integration. The implementation in [19] used discrete components, with capacitor values of up to 51 pF.

Most of the digital phase shifting techniques that are available in the literature have used both counters and delay lines together in order to achieve a high delay resolution with a simple and efficient design [15,18–22]. For example, using this approach, the authors in [18] have managed to implement a phase shifter with a 19-bit dynamic range using a 62.5 MHz reference clock. Even though their target frequency was 1.91 kHz (used for ultrasonic imaging), a 1 GHz reference clock would have been needed for a purely counter based implementation.

**Figure 9.**
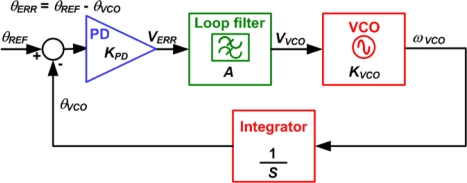
Block diagram of a linearized PLL.

A more sophisticated, although slightly more complex, method to achieve phase shifting is using a phase-locked loop (PLL) [23–26]. The theoretical operation of a PLL can be explained using the block diagram in Figure 9 where the phase error (*θ*_ERR_) can be obtained as:

(2) $$\theta_{\mathrm{ERR}}=\frac{\omega_{\mathrm{VCO}}-\omega_{0}}{AK_{\mathrm{PD}}\;K_{\mathrm{VCO}}}$$

*ω*_0_ is the free running (initial) frequency of the voltage-controlled oscillator (VCO), and *K_VCO_*, *K_PD_*, and *A*, are the gains of the VCO, the phase detector (PD) and the loop filter, respectively. When the PLL is locked at steady state, *ω_VCO_* becomes equal to *ω*_0_ and the phase error is equal to zero. If a DC control voltage (*V_C_*) was subtracted from *V_ERR_*, Equation (2) will change to:

(3) $$\theta_{\mathrm{ERR}}=\frac{\left(\omega_{\mathrm{VCO}}-\omega_{0}\right)+AK_{\mathrm{VCO}}\;V_{C}}{AK_{\mathrm{VCO}}\;K_{\mathrm{PD}}}$$

and in this case, when the PLL is locked, the phase error is equal to:

(4) $$\theta_{\mathrm{ERR}}=\frac{V_{C}}{K_{\mathrm{PD}}}$$

which introduces a non-zero phase shift that depends on the control voltage *V_C_* [23]. However, since the phase error also depends on the gain of the phase detector, the type of phase detector used will affect the range and linearity of the phase control.

Due to component tolerances, non-zero phase error can exist even without applying a control voltage, as well as variations across the different phase shifters in the array. This requires sensitivity and offset adjustment across all the phase shifters in the array [23]. More accurate phase shifting can be achieved using injection-locked (or coupled) PLLs [26] or VCOs [24].

**Figure 10.**
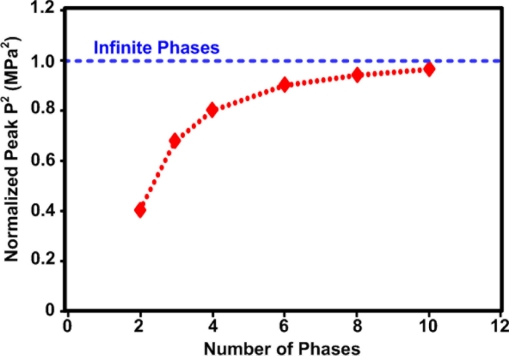
The impact that the number of phases used in a HIFU phase array has on the peak pressure amplitude square (P^2^), simulation reproduced from [20].

Finally, it is also worth mentioning that in order to reduce the number of amplifiers used in the system, a discrete number of phases can be generated and multiplexed to the array elements rather than having full phase control for each element [20]. The simulated impact of reducing the phase resolution can be seen from Figure 10 [20]. This shows that using only four phases will be sufficient to achieve 80% of the power.

## 4. Output Stage and Power Amplifiers

The output stage is the last block before the transducer and is one of the most important blocks in the HIFU system. This is mainly due to the large power and high efficiency requirements. The main function of the output stage is to boost the power of the radio frequency (RF) signal to the required level in an efficient manner prior to transmission through the transducer. In addition to heat generation, a large increase in temperature due to low efficiency can result in a significant variation in output power due to increasing the junction temperature of the active devices. The increase in the junction temperature can be obtained by multiplying the dissipated power by the thermal resistance (°C/W). Thus, the maximum junction temperature sets a limit on the maximum dissipated power in the device to ensure safe operation. The most temperature dependent parameters of a MOSFET device are the effective mobility and the threshold voltage, both of which result in variations in the drain-source current. The temperature dependence of these parameters is given by [27]:

(5) $$\mu (T)=\mu \left(T_{r}\right){\left(\frac{T}{T_{r}}\right)}^{-k_{3}}$$

(6) $$V_{T}\;(T)=V_{T}\;\left(T_{r}\right)-k_{4}\;\left(T-T_{r}\right)$$

where *μ* is the mobility, *V*_T_ is the threshold voltage, *T* is the absolute temperature, *T*_r_ is the room temperature, *k*_3_ is a constant with values ranging from 1.2 to 2.0 and *k*_4_ is in the range of 0.5 mV/K to 3 mV/K. By substituting Equations (5) and (6) in the I–V equation of a MOSFET device, the current-temperature dependence could be obtained.

Although the output stage can be a typical amplifier such as an operational amplifier, it is apparent from the above mentioned reasons that it is desired to have only high efficiency power amplifier (PA) circuits at the output stage. An operational amplifier, for example, will have an efficiency that is below 5% and will continuously consume power, even when no signal is being transmitted. What makes a PA different from any other amplifier, such as a voltage amplifier (operational amplifier) or current amplifier (operational trans-conductance amplifier), is the way the input and output impedances are matched. A typical voltage amplifier provides a very high input impedance (∼mega to tera ohms) and a very low output impedance (∼few tens of ohms) to allow for maximum voltage transfer. On the other hand, a typical current amplifier provides a very low input impedance and very high output impedance. The input of a power amplifier is matched to satisfy the maximum power transfer theorem, having at the best case a 50% power transfer from the source to the amplifier’s input. This loss is acceptable at the input stage since the power level is low. At the output stage however, the maximum power transfer theorem cannot be used and more efficient techniques, used commonly in PAs, are needed. In HIFU, pulsers [22,28,29] or PAs [16,19,21,30–33] can be used for the output stage. Here, we will focus mainly on PAs since they can operate from lower supply voltage levels that are more suitable for portable systems.

PAs are categorized based on their historical appearance, hence the alphabetical classification. The main classes of operation can be divided into two parts, which are the current-mode and the switch-mode, also known as linear-mode and non-linear-mode, respectively. Here, we also add a third category of power amplifiers, which is referred to as lock-mode, and may be more suitable for HIFU applications.

Current-mode PAs are usually less efficient than switch- and lock-mode PAs, however they offer higher linearity. They can provide a theoretical minimum efficiency of 50% that trades off with linearity and output power as increased. Since achieving high efficiency is the most concern in HIFU, linearity can be sacrificed, which is common in biomedical applications [27]. For this reason, most PAs implemented for HIFU do not use linear current-mode topologies. Some designs [19,33] however, have used current-mode PAs, although less efficient, and achieved output power control by controlling the bias of the preamplifier stage with the output of a digital-to-analog converter (DAC) that was controlled by a microcontroller [33]. Having a gated power supply that switches off the output stage when no signal is being applied, can help increase the efficiency.

In switch-mode PAs, the active device operates as a low-resistance (ideally zero) active switch. These classes usually provide higher efficiency than current-mode PAs; however the linearity is usually sacrificed since there is no direct amplitude relationship between the input and output signals, hence they are also referred to as non-linear power amplifiers. The concept of not having an amplitude relationship between the input and output signals may be confusing to designers who are not familiar with non-linear power amplifiers, since this defies the idea of “amplification”. The concept is to generate an output signal that can follow the input signal in phase and frequency, while having a higher power level. Non-linear PAs were found attractive for HIFU systems due to their high efficiencies [16,22,21,30,31]. Mainly, PA classes D and E can be used for HIFU.

**Figure 11.**
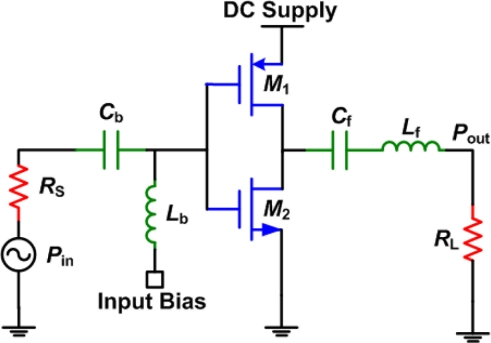
Basic schematic of a class-D power amplifier.

Class-D PAs use two transistors operating as switches, as shown in Figure 11. It is basically a CMOS inverter that generates an output square waveform, which follows the input signal’s phase and frequency. Since the output waveform is square shaped, the output is associated with a filter that passes only the fundamental sinusoidal waveform. The efficiency is affected by the switching time and the on-resistance of the transistors. This class has an advantage of very low, ideally zero, power dissipation and high power capabilities. In HIFU, the transducer itself acts as a very narrowband filter, which can replace the output filter shown in Figure 10. Also, if more than 1 watt of output power is required, an output impedance transformation network is necessary. In the case of multi-element phased arrays, where low output power levels per transducer are required, the output impedance of the PA can be designed to directly match the impedance of the transducer. Avoiding the output matching circuit will be very beneficial in terms of increasing efficiency, avoiding heating in matching elements and reducing the size of the circuit especially since the elements can be large at these frequencies. Matching however, plays an important role since all elements in the array will not have an exactly equal impedance. Also, it is easier to measure the power delivered to a perfectly matched load since there will be no power reflections and only the forward power needs to be monitored [16]. Finally, when digital systems are used to generate the output signal, a square wave will be used to drive the PA, which eliminates the need to bias the input of the transistors M_1_ and M_2_. At the frequencies used for HIFU, it is possible to achieve efficiencies close to 99% with active devices that have an ultralow on resistance [30].

**Figure 12.**
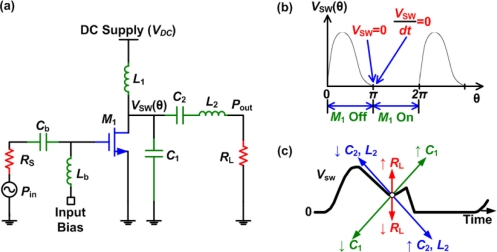
**(a)** Basic schematic of a class-E power amplifier. **(b)** The drain voltage waveform of an ideal class-E amplifier. **(c)** The effects of adjusting the output network components of a class-E amplifier [27].

Class-E power amplifiers were first presented by Sokal and Sokal in 1975 [34] and have recently gained more attraction with the expiry of their patent [35]. This class has an ideal efficiency of 100%. It has a similar configuration as class-D but uses only one transistor that is also operating as a switch, as shown in Figure 12(a).

The pull-up network, which is a PMOS device in class-D power amplifiers, is removed in class-E power amplifiers to improve the efficiency by avoiding the power loss in the PMOS device. To provide the high signal, in place of the PMOS device, an *RFC* inductor (*L*_1_) is used together with a parallel capacitor, *C*_1_. The output waveform of a class-E power amplifier is shown in Figure 12(b), before filtering. The basic criteria to operate a class-E power amplifier are as follows [36]:

1. The voltage across the switch (active device, *M*_1_) at off-mode should not rise until after the transistor turns off.
2. The voltage across the switch should go back to zero immediately before turn-on.
3. The slope of the switch voltage should be zero at turn on (soft-switching).
4. An amplifier with these three criteria is called an “optimum class-E” amplifier, whereas if one of them is missing, it is called a “suboptimum class-E” amplifier [27].

The design equations needed to calculate the output power (*P_out_*) and the value of capacitor *C_1_* can be derived, assuming an ideal *RFC* inductor (*L*_1_), as [27]:

(7) $$P_{\mathrm{out}}=\frac{{8V}_{\mathrm{DC}}^{2}}{R_{L}\left(\pi^{2}+4\right)}$$

(8) $$C_{1}=\frac{{8V}_{\mathrm{DC}}}{\pi \left(\pi^{2}+4\right){\omega R}_{L}}$$

The output filter (*C*_2_, *L*_2_) passes only the fundamental component resulting in a sinusoidal output waveform that is synchronized in phase and frequency with the input. The same points made previously regarding the output filter for the class-D PA are valid here. Also, similarly to class-D PAs, class-E PAs are more efficient when driven by a square wave.

When considering all the non-idealities that exist in the circuit, such as the non-linear output capacitance of the active device, the finite *RFC*, the non-zero on-resistance of the active device and the finite *Q* of the output filter, the output waveform of the circuit can be fine tuned through simulations to ensure optimum class-E operation, shown in Figure 12(b). Figure 12(c) shows how the component values affect the shape of the output waveform. When considering the on-resistance (*R_on_*) of the active device, the drain efficiency can be obtained as [27]:

(9) $$\eta_{\mathrm{drain}}\approx \frac{1}{1+1.4\frac{R_{\mathrm{on}}}{R_{L}}}$$

Equation (9) indicates that it is easier to achieve a high efficiency when *R_L_* is much larger than *R_on_*. This makes using a class-E PA difficult for higher power implementations, since a very low on resistance switch is needed. For moderate output power per element phased arrays for example, an efficiency of above 93% can be achieved with an active device that has a 10 Ω on-resistance that is matched to a 200 Ω transducer.

**Figure 13.**
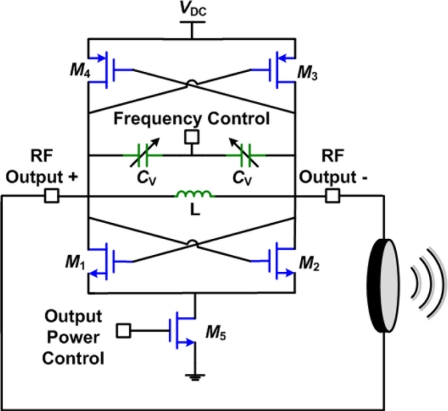
Schematic of a direct-modulation transmitter.

Since most phased array systems involve some form of frequency synthesis, either using a PLL or a function generator and digital dividers, another approach can be taken to generate the HIFU output that does not rely on the use of power amplifiers. High power, high efficiency, VCO and PLL direct modulation transmitters can be used as the output stage [27]. Figure 13 shows an example of a power voltage controlled oscillator (PVCO) used for the output stage.

The circuit consists of a simple negative-*g_m_* cross-coupled pair oscillator with voltage controlled varactors. This circuit can be designed to deliver output levels as high as 50 mWs with close to 50% efficiencies [27]. In order to track the free running output frequency and have better control of the circuit, the PVCO can be used as part of a PLL. The efficiency can be increased even further using a class-E PVCO [27].

Finally, it is worth mentioning that most switch-mode PAs require a large input drive. Current-mode PAs also only achieve their maximum efficiency at the compression point, where the gain is minimum and the input drive is highest. In order to reduce the input drive requirement of the output stage in an HIFU system, lock-Mode PAs can be used. Since the output signal of a non-linear PA has a fixed power that is in theory not a function of the input signal, a non-linear PA can be considered an oscillator whose phase and frequency follow a reference signal (the input).

The input power required to drive a non-linear amplifier to “create” an output signal is much larger than the input power required to “influence” an existing signal. If the PA was biased on the verge of oscillation, or somehow an oscillator was used at the output, the required input drive will actually be lowered, since the output signal will in a sense already exist and it will take less effort to lock it than to create it. This concept is known as mode-locking and has recently gained importance in switch-mode power amplifiers [27]. Lock-mode power amplifiers usually have a very high power gain when compared to both current-mode and switch-mode power amplifiers. Their achievable efficiency however, will depend on the type of PA used within the lock-mode circuit.

**Figure 14.**
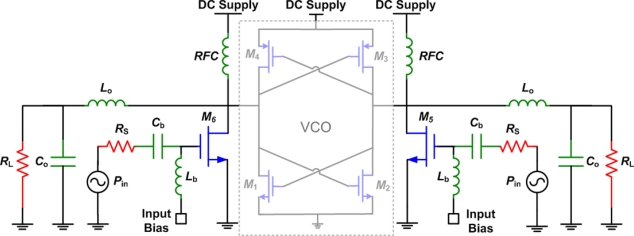
Differential class-E power amplifier with mode-locking, reproduced from [40].

Most mode-locking works [37–40] use harmonic injection-locking, where the input and output frequencies are equal. Mode-locking can also be subharmonic, when the output frequency is higher than the input frequency or superharmonic, when the output frequency is lower than the input frequency. In [40], a cross-coupled differential negative-*g_m_* VCO was used as an oscillator to provide the output signal to a differential class-E power amplifier. Figure 14 shows the basic idea proposed in [40].

Here we propose a differential superharmonic injection-locked frequency divider (ILFD) to operate as a lock-mode power amplifier for moderate power, HIFU applications. ILFDs are based on injection-locked oscillators (ILOs), which are free running oscillators that can lock to the phase and frequency of an injected signal. The main advantage of the proposed approach is that it can achieve a very high power gain from a single stage that is very efficient in terms of silicon die area as well as power.

**Figure 15.**
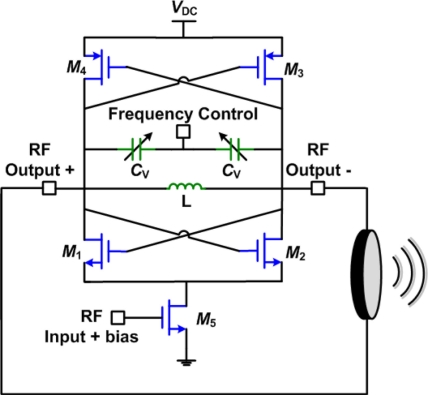
Basic schematic of the lock-mode power amplifier [27].

Figure 15 shows the basic schematic of the proposed lock-mode power amplifier [27]. The difference between this design and the one shown previously in Figure 13 is in the sizing of transistor *M*_5_. In the direct modulation transmitter case, which has no RF input, transistor *M*_5_ should be designed to have a long channel to be used as a current source. In the case of ILPA, transistor *M*_5_ is the input stage of the PA and should be designed for high speed, high gain operation. This makes the circuit operate in a voltage limited regime, which is supply dependant, with no constant current source biasing. A cascoded tail current source transistor can be added, however, it will cause a slight reduction in the gain provided by transistor *M*_5_.

In this design the incident frequency is divided by two. It could be argued that this presents an obstacle to practical application since it requires doubling the operating frequency of the previous RF stages. However, this is not an issue in HIFU since the previous phase comes from an already higher frequency that is used for frequency synthesis and phase control. Also, the input power required to achieve mode-locking is very small, the input drive is greatly reduced compared to a traditional power amplifier. This allows a doubling of the operating frequency of the previous stage without significantly increasing the power consumption in that stage. A small signal gain of over 30 dB can be achieved by a single stage of this design, with a 50% efficiency in a small circuit area. Also, similarly to the direct-modulation transmitter, the efficiency can be increased by using a more efficient class-E PVCO [27].

## 5. Conclusions

This paper has reviewed several techniques used for driving phase array high intensity focused ultrasound (HIFU) systems. The ultimate goal when designing a large array size HIFU system would be to have a compact solution that can fit on the back of the ultrasound probe. Although, a few attempts to miniaturize the driving system exist [21,31,32], they are either designed to drive a single element or are still quite large. Therefore, it is desirable to design a compact, small size driving system using integrated solutions. Switch-mode, injection-locked, or direct-modulation high efficiency output stages should be used. Switching between 4–8 phases rather than generating and controlling each element’s phase individually, should also be considered for very large arrays [20].
